# Editorial: Advances of Targeted Therapy in Gynecologic Malignancies

**DOI:** 10.3389/fonc.2022.887773

**Published:** 2022-04-05

**Authors:** Haifeng Qiu, Kun Song, Lei Wang, Chunxiao Zhou

**Affiliations:** ^1^ Department of Gynecology, The First Affiliated Hospital of Zhengzhou University, Zhengzhou, China; ^2^ Henan Province Engineering Research Center of Fertility Preservation in Gynecologic Tumors, Zhengzhou, China; ^3^ Department of Obstetrics and Gynecology, Qilu Hospital of Shandong University, Jinan, China; ^4^ School of Pharmaceutical Sciences, Zhengzhou University, Zhengzhou, China; ^5^ Division of Gynecologic Oncology, University of North Carolina at Chapel Hill, Chapel Hill, NC, United States; ^6^ Lineberger Comprehensive Cancer Center, University of North Carolina at Chapel Hill, Chapel Hill, NC, United States

**Keywords:** ovarian cancer, endometrial cancer, cervical cancer, targeted therapy, immunotherapy

## Introduction

Gynecologic malignancies, a heterogeneous group of female reproductive system tumors (including ovarian, endometrial, cervical, vaginal and vulvar cancer), are the second most commonly diagnosed female cancers worldwide (1). Among these cancers, cervical cancer is the most common malignancy of the female genital tract, followed by endometrial cancer and ovarian cancer. Most patients with early stage gynecological cancers are cured with surgery alone or a combination of surgery, radiation and chemotherapy. However, patients with advanced, recurrent, or metastatic disease lack effective therapeutic options and often have poor prognosis despite appropriate managements. Therefore, there is an urgent need to develop new-targeted therapies based on the molecular features of gynecological cancers and their microenvironment.

## Ovarian Cancer

In addition to these genetic aberrations, epigenetic regulation of key genes has been reported in ovarian cancer (2). Two studies on this Research Topic described the roles of DNA methylation in ovarian cancer. First, Li et al. detected that down-regulation of MGRN1 caused by hypermethylation was significantly associated with platinum resistance and worse survival in high-grade serous ovarian cancer (HGSOC) patients. Furthermore, knockdown of MGRN1 notably reduced the sensitivity of cell to cisplatin *in vitro*. Second, in a cohort of 16 matched primary-recurrent advanced serous epithelial ovarian cancers, Visco et al. found a stronger correlation between Claudin-1 (CLDN1, a tight junction protein) expression and methylation in recurrent versus primary ovarian cancer at multiple CpG sites, which was stronger in patients with poor prognosis (overall survival < 3 years). In addition, another study demonstrated that ALOX5AP was significantly up-regulated in serous ovarian cancer and was associated with poor prognosis. Further analysis revealed that ALOX5AP was predominantly overexpressed in these immunoreactive cases and correlated with immunocyte infiltration, especially M2 macrophage polarization. Their findings suggest that ALOX5AP acted as a prognostic predictor for serous ovarian cancer and deserved further exploration in clinical studies as an immunotherapeutic target.

Compared to the well-defined effects of conventional chemotherapeutic drugs such as platinum and paclitaxel), exploring novel targets and designing efficient regimens have become crucial for the treatment of ovarian cancer. In this Research Topic, two candidate therapeutic targets were reported. Wei et al. demonstrated that targeting ACLY can inhibit PI3K/AKT pathway and activate the AMPK/ROS pathway, thereby inhibiting the tumor growth and reversing the acquired chemo-resistance of ovarian cancer. Another study found that BTG2, a member of anti-proliferative gene family, induced G1/S phase arrest and inhibited migration by targeting Cyclin D1, CDK4, p-AKT, and p-ERK MMP2/9 and MMP-9, suggesting BTG2 as a potential therapeutic candidate for ovarian cancer. Tran et al. evaluated the efficacy of SPR965, a dual inhibitor of PI3K/mTOR pathway, in serous ovarian cancer cell lines and a transgenic mouse model of ovarian cancer. They confirmed that SPR965 significantly inhibited the proliferation, adhesion and invasion in ovarian cancer cells and the mouse model, indicating SPR965 may have potential to treat ovarian cancer.

PARP inhibitors are now well known and widely used in ovarian cancer patients, while more precise molecular markers are needed to select patients and monitor treatment response (3). Nakanishi et al. demonstrated that a lower NLPN scores [recurrent neutrophil-lymphocyte ratio (rNLR) × number of previous regimens] was associated with favorable outcomes of olaparib. However, these patients with high rNLR should start the olaparib treatment as early as possible to obtain a lower NLPN score. This may provided a novel strategy to predict the effects of PARP inhibition regardless of BRCA or HRD status.

## Cervical Cancer

The persistent infection with high-risk HPV is a well-established risk factor of cervical cancer. Extensive screening with HPV testing and liquid-based cytology has greatly reduced the incidence of precancerous lesions and invasive cervical cancer over the past few decades (4). For these early-stage tumors, (radical) hysterectomy ± adjuvant chemo-radio-therapy usually yields satisfactory outcomes, but the situation is quite different in advanced or recurrent cervical cancer (5). In addition to conventional approaches, researchers are now focusing on investigating novel therapeutic targets for cervical cancer. In this Research Topic, Jiang et al. found that TIPE1 can promote the growth of cervical cancer through inhibiting cellular apoptosis and facilitating chemo-resistance in a wild-type p53-dependent manner. Liu et al. demonstrated that CD155 can form a complex with AKT to activate the downstream mTOR/NF-κB pathway in cervical cancer cells, and targeting CD155 significantly inhibited autophagy and apoptosis *via* blocking the AKT/mTOR/NF-κB signal pathway. Wang et al. provided the first direct evidence of crosstalk between NGF and Hippo signaling pathways in cervical cancer progression. They proposed the dual blockade of NGF and Hippo signals represented a novel therapeutic strategy. CircRNAs played imported roles in cancer and several of them have been proved as candidates for targeting therapy. Herein, Liao et al. revealed that low expression of hsa_circ_0107593 was negative correlated with tumor diameter, FIGO stage, and myometrial invasion in cervical cancer. *In vitro*, hsa_circ_0107593 serves as a sponge of hsa-miR-20a-5p, hsa-miR-93-5p, and hsa-miR-106b-5p, functioning as a tumor suppressive circRNA.

There are still some controversies in the treatment of locally advanced cervical cancer (6). Dang et al. evaluated the efficacy of neoadjuvant chemotherapy combined with brachytherapy in a panel of 183 stage IB2 and IIA (FIGO 2009) cervical cancers. Neoadjuvant chemotherapy plus brachytherapy improved PFS by 29% and decreased the need for postoperative adjuvant pelvic radiotherapy. Adenosquamous carcinoma of the cervix (ASCC) presented both the characteristics of adenocarcinoma and squamous cell carcinoma and usually requires more attention in the treatment and surveillance. Cui et al. summarized the information on 1142 ASCC patients in a population-based study. They then constructed a nomogram which precisely predicted 3- and 5-year cancer-specific survival, and exhibited more clinical benefits than the FIGO staging system.

In two other articles, the authors focused on the tumor microenvironment and immune status in cervical cancer. Rossetti et al. found that expression increase and p65 NF-κB increased the expression of STAT3 and decreased the expression of p65 NF-κB in circulating leukocytes were related to lesion grade. Inhibition of STAT3 results in slow tumor growth, increased anti-tumor T cell responses and decreased the accumulation of myeloid cells in the spleen in a tumor experimental model. They also found that local and systemic STAT3 and p65 NF-κB expression can serve as progression markers and functional targets for cervical cancer; Chen et al. developed an immune-based prognostic score (IPRS) based on the immune signatures of 296 cervical cancer patients. Multivariate analysis revealed that IPRS was an independent prognostic factor for overall survival (OS) and progression-free survival (PFS). Furthermore, higher IPRS were associated with better survival, which was further validated in the validation set.

## Endometrial Cancer

Although most endometrial cancer was estrogen-dependent, progestin resistance was not uncommon in the clinic (7). Cui et al. demonstrated that Chlorpromazine (CPZ) reversed the progestin resistance by inhibiting PI3K/AKT signal and up-regulating PRB level *in vitro* and *in vivo*. Sequential but not simultaneous treatment with CPZ and medroxyprogesterone acetate (MPA) showed more synergistic effect in suppressing tumor growth endometrial cancer. Therefore, CPZ may be an appropriate option for these patients with progesterone-resistant endometrial cancer. The AMPK signaling pathway as a cellular energy sensor is involved in regulation of the glucose metabolism and has been proved to be a potent target for the treatment of type I endometrial cancer. Roque et al. tested the anti-tumor activity of NT-1044, a new biguanide with higher affinity for the OCT1 and OCT3 transporters in endometrial cancer cells and a transgenic mouse model of endometrial cancer. Through increasing AMPK phosphorylation and decreasing S6 phosphorylation, NT-1014 caused G1-phase arrest, enhanced apoptosis and increased ROS production in endometrial cancer cells. As compared to metformin, NT-1044 exhibited the similar anti-tumor activity in LKB1fl/flp53fl/fl mouse model of endometrial cancer under obese and lean conditions. Cai et al. identified LMTK 3 as a novel therapeutic target for endometrial cancer. They found LMTK3 was mainly over-expressed in the cytoplasm of cancer cells, and knockdown of LMTK3 decreased cellular viability, increased G1-phase arrest and promoted apoptosis in endometrial cancer cells.

## Comprehensive Studies and Others

In order to discriminate the common/exclusive genomic alterations, Guo et al. conducted whole-exome sequencing in 209 cervical, endometrial and ovarian cancer samples. Their results suggested the three cancer types shared a common reprogramming process during early carcinognensis, including PI3K activation, mismatch repair, and defects in ciliary organization, as well as disruption of interferon signaling and immune recognition. While cell-type specific programs were detected in the late-stage of tumor development, ultimately leading to tumor proliferation and migration. Similarly, Alldredge et al. explored the integrative transcriptomic profiles of clear carcinoma originated from ovarian and endometrium. Besides the common comparative analysis, the authors identified out 3,252 differentially expressed genes between PD-L1+/− ovarian clear cell cancers, which were enriched in immune response, cell death, and DNA repair networks As for the immune cells, PD-L1+ tumors had more resting NK cells and memory B cells, while CD8 T-cells, monocytes, eosinophils, and activated dendritic cells were the main cell types in PD-L1− tumors. These findings revealed novel characteristics of clear carcinoma and might be helpful for drugs selection, especially for patients who planned to take immunotherapy.

Besides the original studies mentioned above, there were several reviews in this Research Topic. Reyes-González et al. discussed the frequency of c-MYC deregulation in ovarian cancer and the consequences of its targeting. In another review, Chen et al. focused on the features of endometrioid ovarian cancer, a relative rare form of epithelial ovarian cancer. They compiled the potential mechanisms of endometrioid ovarian cancer, molecular characteristics, and molecular pathological types, which might provide guidance for the stratified management of ovarian cancer.

Overall, these multi-angle articles in this Research Topic presented broad advances in targeted therapy for ovarian, endometrial and cervical cancer. We hope that these valuable findings will contribute to further development of effective targeted therapies for the benefits of patients with gynecologic cancers

## Author Contributions

All authors listed have made a substantial, direct, and intellectual contribution to the work and approved it for publication.

## Conflict of Interest

The authors declare that the research was conducted in the absence of any commercial or financial relationships that could be construed as a potential conflict of interest.

## Publisher’s Note

All claims expressed in this article are solely those of the authors and do not necessarily represent those of their affiliated organizations, or those of the publisher, the editors and the reviewers. Any product that may be evaluated in this article, or claim that may be made by its manufacturer, is not guaranteed or endorsed by the publisher.
